# Organized crime requires dynamic decision making

**DOI:** 10.3389/fpsyg.2024.1205135

**Published:** 2024-02-02

**Authors:** José Kerstholt, Bas Keijser, Guido Veldhuis, Eefje Smits-Clijsen

**Affiliations:** ^1^Netherlands Organisation for Applied Scientific Research (TNO), Soesterberg, Netherlands; ^2^University of Twente, Enschede, Netherlands

**Keywords:** organized crime, decision making, systemic approach, decision support, training

## Abstract

It is extremely hard to successfully fight organized crime, not only because there are ambiguous and complex interactions between factors and actors, but also because organized crime is volatile and adaptive to changing conditions. This paper argues that, in addition to acquiring knowledge on criminal processes and behavior, we also need a better understanding of organizational decision-making processes to select the most effective and sustainable interventions aimed at organized crime. To date, one-shot decisions from a single organizational perspective are predominant in fighting organized crime. This type of decision often ignores the system response to the intervention. There is a need to acknowledge the dynamic nature of criminal behavior and networks, and to take that into account in the design of an intervention strategy. Such an approach entails a sequence of interdependent steps, iteratively applied, to reach sustainable effects. However, this way of decision making does not come naturally for most people. This paper concludes that investments should be made in training and decision support for teams fighting organized crime.

## Introduction

It is extremely difficult for crime fighting teams to study and understand organized crime. The secretive nature of criminal groups is one major challenge, which makes it difficult to obtain reliable information about their activities. Another challenge is posed by criminals not operating in a social vacuum; there is an interplay between intergroup and intragroup factors across social networks (Roks et al., 2022). The importance of secrecy in turn reinforces a tendency to only rely on strong, well-known, social bonds amongst co-offenders and to isolate themselves from others. At the same time, criminals also need connections in the licit social domain to execute their criminal activities. For example, to smuggle cocaine, port employees are bribed (Roks et al., 2021) and to launder money legal businesses are setup (Malm and Bichler, 2013). Relating to these licit networks, criminals secure a ‘wall of silence’ by engaging in a range of strategies: keeping parties either ignorant, financially satisfied or fearful (Roks et al., 2022).

Another complicating factor in understanding organized crime is that it develops dynamically over time, it is not a static phenomenon. Criminals and crime groups are highly resilient, because of environmental factors and individual and organizational features (Ayling, 2009). They quite flexibly adapt to new circumstances, such as new locations, forms of crime, modus operandi or different partners. Crime displacement in time, place and modus operandi is a long-known effect (Repetto, 1976). Therefore, it is broadly recognized in criminology that the phenomenon should be studied as a system of interrelated actors and factors.

### Systemic thinking for organized crime

As pointed out by Richardson (2011), a systems approach to crime would take an endogenous viewpoint. An endogenous viewpoint seeks to explain the dynamics of organized crime over time as the result of the interaction of actors and factors within a broad system. Well-known systems perspectives in criminology emphasize, e.g. amongst others the relevance of endogenous, reinforcing and balancing causal mechanisms to describe system dynamics inherent to criminal phenomena such as coca cultivation (Jaén and Dyner, 2014). Relatedly, a criminal network active in illicit drug smuggling can be considered a system too, which is custom in network analysis and related approaches to modelling and simulating criminal networks (Burcher and Whelan, 2018). In our view crime teams should apply systems thinking to identify and understand systems, explore their behaviors, so that they start with a clear problem definition and from there devise changes to the system to be effectuated through interventions (Arnold and Wade, 2015). Creating a system representation of a crime problem requires the right mindset and thinking processes (Cabrera et al., 2021).

To sustainably fight organized crime, we therefore do not only need insight into distinctive forms of organized crime and criminal networks through applying a systems approach, but also knowledge on how to make decisions to attain maximal effects in reducing, disrupting or containing organized crime.

## Dynamic decision making

### Observations from practice in the Netherlands

Over the past five years we have been involved in applied research supporting crime fighting teams. Activities involved observational research into challenges in analysis and collaboration, support to analysis and group decision-making through group model building, as well as participation in crime fighting teams as ‘scientists-on-the-job’. One of the main conclusions from this work is that teams largely have a linear way of thinking: they see simple one-directional chains of cause and effect instead of a multicausal view involving feedback effects, time delays and non-linearities. This causes teams to focus on a one-shot solution for the problem. For example, when arresting well-known producers of synthetic drugs, production is disrupted in the short term. However, when there are no other experienced cooks available, less experienced individuals may spring into action resulting in physical safety risks. Crime fighting teams generally do not take the possibility of such criminal actor responses into consideration.

These observations are in line with research on dynamic decision making which has shown that humans perform poorly in dynamic decision making tasks, even when the task is reduced to its simplest form and after lots of practice (Diehl and Sterman, 1995; Kerstholt and Raaijmakers, 1997; Gonzalez et al., 2017). People find it difficult to understand dynamic systems. Several biases are mentioned that may explain this effect. One of these biases is that people assume that the relation between two variables is linear and unidirectional (Sterman, 1989; Diehl and Sterman, 1995). Another one is that people tend to focus on specific details of the system, rather than on the system as a whole (Kerstholt and Passenier, 2000; Fischer and Gonzalez, 2016). To understand system behavior several system thinking skills are necessary, Richmond (1993) has identified seven skills. The need to step back and oversee the situation at hand, is called forest thinking. Forest thinking is the ability to rise above the local trees, to rise above the details, to see trends and anticipate future states. Furthermore, dynamic thinking is “to see and deduce behavior patterns rather than focusing on, and seeking to, predict events” (Richmond, 1993: 122). This skill is necessary to recognize a problem’s dynamic character.

As it is realized that repressive interventions are not sufficient to reduce organized crime (Levi and Maguire, 2004; Kleemans and Soudijn, 2017), collaboration with non-law enforcement partners is complementarily used to attain sustainable effects. Organizations such as merchant banks, tax authorities and municipalities have different information positions and different interventions. Merchant banks can, for example, detect large deposits of money, tax authorities can identify tax evasion and municipalities can check criminal records and close down businesses or withdraw permits. By collaborating, a broader view and set of interventions to disrupt criminal activities becomes available (Klerks, 2016). However, despite its potential, including multiple players to the team does not automatically result in systemic thinking.

A first challenge is that in a collaboration of multiple organizations each has their own goals and interests that may not be compatible with each other. If interventions are chosen from a systemic perspective, the team needs a shared set of goals, focused on the problem to be solved rather than the organizational goals of individual parties (Klerks, 2016). Relatedly, performance indicators are often not defined at the level of shared goals. As noted by Zürcher et al. (2023), performance indicators for the police with in the Netherlands are still typically based on unilateral goals relating to outputs, such as number of investigations completed, and arrests made. To complementarily apply a different, systemic, mindset it is good to also link performance measurements to shared goals and outcomes, next to individual organizational goals and outputs (Moore and Braga, 2003).

### Why dynamic decision making is needed

To select sustainable interventions in a process of dynamic decision making, there is a need to have more insight into the relations between the relevant actors and factors across various domains, to get a good understanding of the system at hand. When interventions selected in a dynamic decision making process are focused on these systemic elements, the probability increases that interventions lead to sustainable effects.

A broad overview of the criminal system in dynamic decision making is also needed to both forecast unwanted side-effects and to recognize them after an intervention has been applied. Disrupting drug trafficking in a specific sea harbour may, for example, result in criminals exploring the next sea harbour or a new modus operandi to pick up packaged drugs (Paoli, 2016; Roks et al., 2021). In addition, side effects can also occur across domains of society: interventions to disrupt trust in criminal networks might backfire when it also leads to increased violence in residential areas. Thus, it is important to describe all relevant system domains as well as their mutual relationships to understand the context of criminal behavior.

### How can decision making be improved?

In the present paper we argue that tackling organized crime needs a different type of decision-making than commonly seen. Even though viewing organized crime as a complex dynamic system has become custom in criminology, this view is mostly not adopted by crime fighting teams. One-shot interventions are insufficient; an intervention strategy is needed that considers dependencies between interventions and related factors, including available intelligence, actions, effects and unwanted side effects.

As described above, however, this is not a natural way of making decisions, implying that conscious investments are needed to shift to a different way of thinking. Dynamic decision making is hard for human beings, and performance generally low. Below, we discuss two requirements to make a shift toward systemic thinking and dynamic decision making. First, a mindset is needed to conceptualize specific signals of crime in a wider context. In addition, support is needed to facilitate actual decision-making concerning complex, dynamic systems.

#### Mindset

Humans typically think in a linear way. If, for example, there is an indication of money laundering, a natural reaction is to activate tax authorities to do an investigation. Our mind naturally links a direct solution to the problem at hand. As argued above, however, it is beneficial to also have a wider view on the system and to apply, e.g., Richmond’s systems thinking skills (Richmond, 1993). Such a view would allow for the identification of causal roots, potentially leading to more sustainable effects and insight into causal relations to predict potential (side-)effects of interventions. Right from the start of a case this wider context needs to be addressed, as the (first) framing of the problem will define the focus and follow-up actions. After that action can be taken, to subsequently strengthen analysis – mitigating an apparent paradox between analysis and action (Waardenburg et al., 2020). That does not mean that a systemic approach should be used for each case, but it does mean that a choice to take a local perspective should be made deliberately.

Dynamic decision making requires metacognition, the act of monitoring and reflecting on one’s own cognition (Azevedo, 2020; Cabrera et al., 2021). Metacognition supports both critical thinking, the ability to assess the truthfulness of one’s approach (Magno, 2010), and perspective taking, which is a cognitive process of imagining the world from another’s vantage point to understand thoughts, motivations, intentions and emotions (Ku et al., 2015). Metacognitive judgments can be increased through training.

#### Support

To support problem structuring and analysis of crime as a complex system, various methods have been developed, see earlier given examples on system dynamics and criminal network analysis, and Barros et al. (2022). A specific example method of interest is MARVEL (Method to Analyse Relations between Variables using Enriched Loops; Veldhuis et al., 2015).

Causal modelling methods such as MARVEL support the description of causal relations between variables, also taking strength and speed into account. Models are built in sessions with relevant stakeholders, strengthening shared understanding of the system at hand as well as the various perspectives that individual stakeholders have. In addition, the model can be used to (mentally) simulate system effects when specific variables are changed which contributes to the development of a theory of change. Building a model together enhances shared understanding of the situation at hand and serves as the basis for collaborative decision making.

## Conclusion

Given the complex and dynamic character of organized crime, a radically different approach to decision making is required to attain sustainable effects. Rather than solving local, isolated problems crime fighters need a systemic view on organized crime to understand underlying elements, interconnections and system dynamics. Such an approach requires an analysis and intervention strategy consisting of a range of (cross domain) interventions, taking interdependencies and system reactions to interventions into account. However, as our natural way of decision making is more locally oriented, directly mapping a single solution to the identified problem, it is important to invest in training and decision support to succeed in achieving a shift in mindset toward systemic thinking and dynamic decision making. Criminal organizations are resilient and adapt to interventions and new opportunities. To have a chance at effectively fighting organized crime, cross-domain efforts should be combined in designing a broad intervention strategy based on a systemic understanding of organized crime.

## Author contributions

JK wrote the first draft of the paper. BK, GV and ES-C contributed to the main ideas described in the paper. All authors contributed to the article and approved the submitted version.
